# Simplified Vertical Ridge Augmentation in Severely Resorbed Alveolar Ridges Using a Novel Wide-Head Tenting Pole Screw: Clinical and Histomorphometric Analysis—A Case Series

**DOI:** 10.3390/jcm14196772

**Published:** 2025-09-25

**Authors:** Hyung-Gyun Kim, Yong-Suk Moon, Dong-Seok Sohn

**Affiliations:** 1 Department of Dentistry and Advanced General Dentistry, Daegu Catholic University School of Medicine, Daegu 42472, Republic of Korea; hgkim25@cu.ac.kr; 2 Department of Anatomy, Daegu Catholic University School of Medicine, Daegu 42472, Republic of Korea; ysmoon@cu.ac.kr; 3 Department of Dentistry and Oral and Maxillofacial Surgery, Daegu Catholic University School of Medicine, Daegu 42472, Republic of Korea

**Keywords:** vertical ridge augmentation, guided bone regeneration, bone regeneration, screw-guided bone regeneration, ridge augmentation techniques

## Abstract

**Background/Objectives**: Vertical ridge augmentation remains a challenging procedure in alveolar bone reconstruction, with existing techniques often limited by surgical complexity, graft instability, and high resorption rates. This study evaluates the clinical and histological outcomes of a novel vertical ridge augmentation technique using a wide-head tenting pole screw (WHTPS) combined with sticky bone graft material. **Methods**: Five patients with vertical bone deficiencies (6–10 mm) in the maxilla or mandible underwent augmentation using a single WHTPS (rectangular or round wide-head type). Sticky bone was prepared using autologous tooth bone, allografts, or xenografts, combined with fibrin glue and covered with concentrated growth factor (CGF) membranes and/or resorbable collagen membranes. After 5–6 months of healing, the WHTPS was removed, and bone biopsies were taken for histological analysis. **Results**: Radiographic and histological evaluations confirmed successful ridge augmentation in all cases. Newly formed bone ranged from 21.2% to 57.5%. All patients proceeded to implant placement without complications. Radiographic, clinical, and histological assessments consistently showed that new bone formation extended up to the level of the screw head, indicating complete vertical fill of the augmented space. Histology showed well-integrated, mineralized bone with no signs of inflammation. The wide-head tenting pole screw was observed to support stable space maintenance and facilitate surgical handling and favorable outcomes in vertical ridge augmentation. **Conclusions**: In this case series, a single wide-head tenting pole screw appeared sufficient to maintain space and resist soft tissue pressure in wide alveolar bone defects during healing. This case series suggests that the wide-head tenting pole screw technique may be a feasible option for managing severe alveolar bone deficiencies.

## 1. Introduction

The loss of teeth and alveolar bone due to various causes significantly impairs masticatory function and aesthetics, leading to a considerable decline in patients’ quality of life [1]. To address this issue, numerous treatment modalities have been developed, with dental implants becoming the most widely adopted due to their reliable outcomes in both function and aesthetics [2].

However, placing implants in areas with severe alveolar bone defects is often considered a significant clinical challenge due to insufficient bone quality and volume. Vertical ridge augmentation is often needed for implant placement in areas with severe alveolar bone defects. However, the procedure remains challenging due to surgical complexity, graft instability, and variability in bone regeneration, making clinical outcomes difficult to predict [2,3,4]. Various techniques have been used to overcome these challenges, such as guided bone regeneration (GBR), block bone grafting, distraction osteogenesis, sandwich osteotomy, and the tent-pole screw technique [5,6,7,8]. Among these techniques, GBR has gained widespread popularity due to its relatively straightforward surgical approach and the possibility of simultaneous implant placement [3,9]. However, despite these advantages, GBR has its limitations, such as the risk of early membrane exposure and the potential instability of the grafted area [4,10]. Block bone grafting has long been considered the gold standard for reconstructing vertical bone defects. However, it has certain drawbacks, including the unpredictability of wound dehiscence and graft resorption, the need for a donor site, and complications such as fractures, sensory disturbances, hematoma, and infection [11,12]. Intraoral distraction osteogenesis is advantageous for achieving vertical bone augmentation in a relatively short period, along with simultaneous soft tissue expansion in proportion to the extent of bone formation [13,14]. However, this technique has several limitations, including the unesthetic appearance of the distraction device, the risk of fractures, soft tissue dehiscence, infections, and the potential for undesirable direction vectors [15]. The sandwich osteotomy technique can preserve the attached gingiva on the lingual or palatal side, thereby maintaining blood supply to both the graft and the transport segment [16,17]. However, this technique is associated with certain drawbacks, including fractures of the transport segment and soft tissue dehiscence at the graft site [17].

The tent-pole screw technique utilizes screws to create a tent-pole effect, allowing for space maintenance beneath the soft tissue. This technique offers the advantages of being applied to a single surgical site and being relatively straightforward and technically simple [18]. However, the small size of the screw head limits the amount of bone regeneration as the distance from the screw increases, necessitating the use of multiple screws in cases of extended edentulous spans. Furthermore, the inability to perform bone grafting and implant placement simultaneously requires an additional surgical procedure, thereby prolonging the edentulous period, which is a significant limitation of this technique [19].

Recently, a vertical ridge augmentation technique utilizing a wide-head tenting screw has been introduced in clinical practice to simplify the surgical procedure, reduce operating time, and prevent the collapse of bone graft material caused by the contraction of the soft tissue matrix during the healing period. Compared to traditional tent-pole screws or titanium mesh, the wide-head tenting pole screw (WHTPS) offers improved space maintenance due to its wide head design, which may also reduce graft micromovement and enhance regenerative predictability. While conventional methods are often associated with complications such as titanium mesh exposure, infection risk, soft tissue dehiscence, and graft instability [18,19,20], the WHTPS could help overcome some of these limitations. However, its histological outcomes have not been thoroughly documented in the existing literature [21]. This report aims to present the clinical and histological outcomes of vertical ridge augmentation using a single WHTPS, commercially known as Sohn’s Bone Builder (SBB^®^, Biotem Co., Hanam-si, Gyeonggi-do, Republic of Korea), and sticky bone graft material in cases of extensive vertical bone defects.

## 2. Materials and Methods

### 2.1. Study Design

#### 2.1.1. Inclusion and Exclusion Criteria

The inclusion criteria for this case series were as follows:

(1) Adult patients aged 18 years or older.
(2) Presence of vertical alveolar bone defects measuring ≥6 mm in height that require augmentation before implant placement.

Patients were excluded if they met any of the following criteria:

(1) Presence of uncontrolled systemic diseases like diabetes mellitus or osteoporosis.
(2) History of chemotherapy or radiotherapy involving the head and neck region.

#### 2.1.2. Classification of Hopeless Teeth

Hopeless teeth were identified based on the classification proposed by Kwok and Caton [22], which defines them as teeth with insufficient potential for periodontal stability requiring extraction. Clinical indicators included severe attachment loss, Grade III mobility, and furcation involvement. This classification was validated in a recent retrospective study reporting a five-year survival rate of only 17.7% for hopeless teeth [23].

#### 2.1.3. Surgical Procedure and Bone Biopsy Protocol

In cases requiring tooth extraction, teeth diagnosed as hopeless based on the criteria outlined in Section 2.1.2 were extracted. The extracted teeth were subsequently processed into graft materials. A standard crestal incision was made, followed by full-thickness flap elevation. The defect site was meticulously debrided to remove all soft tissue and granulation tissue. A WHTPS was then placed in the area requiring augmentation to maintain vertical space against soft tissue pressure, with a minimum of 3 mm engagement into native bone to achieve primary stability; depending on the size of the vertical defect and the residual bone height, screws ranging from 6 mm to 10 mm in length were used.

Autologous fibrin glue was prepared from the patient’s venous blood and mixed with various graft materials, including autogenous bone, allograft, xenograft, and tooth-derived grafts, to form a cohesive sticky bone matrix. This mixture was grafted around the WHTPS up to the height of the screw head. A resorbable collagen membrane was placed over the grafted site, and tension-free primary closure was achieved via periosteal releasing incisions.

Healing periods varied among cases; however, in most cases, implant placement was performed approximately 5 months after grafting. At the time of implant placement, a core biopsy was obtained from the planned implant site using a trephine bur with a 3.0 mm inner diameter and a cutting length of 7.0 mm.

#### 2.1.4. Histological Processing and Analysis

Biopsy specimens were fixed in 10% neutral buffered formalin for 24 h and decalcified in 10% formic acid for 7 days. The decalcified specimens were embedded in paraffin and sectioned at a thickness of 5 μm. Each specimen was divided into five sections based on location. Sections were stained with hematoxylin-eosin and Masson’s Trichrome and examined under light microscopy for new bone formation and soft tissue characteristics. Histomorphometric analysis was conducted using the AxioVision SE64 software, version 4.9.1 (Carl Zeiss, Oberkochen, Germany), and five images per specimen were analyzed. The percentage of new bone and residual graft material was calculated as the area ratio relative to the total tissue area, with the final percentage determined as the average of the five measurements. In Masson’s Trichrome stain, newly formed bone was identified by blue coloration and measured accordingly.

### 2.2. Study Outcomes

The primary outcome of this study was vertical bone regeneration, measured as the percentage of newly formed bone beneath the WHTPS using histomorphometric analysis of biopsy specimens obtained during implant placement.

The secondary variables included:

(1) Radiographic evidence of vertical ridge augmentation confirmed by CBCT and periapical radiographs, showing vertical bone gain up to the screw head level.
(2) Clinical feasibility and surgical success, defined as uneventful healing and the removal of the WHTPS without complications.
(3) Histological quality of the regenerated bone, including the presence of mature lamellar bone, integration with graft material, absence of inflammation, and presence of active osteoblasts and osteocytes.
(4) Graft containment and membrane stability depend on whether the sticky bone graft and CGF or collagen membrane remain in place without collapsing or requiring additional fixation.

These outcomes were recorded in all five cases using clinical photographs, radiographic data, and histological analysis

### 2.3. Study Design Limitation

This study was conducted as a retrospective case series without a control group. Although this design allowed for detailed clinical and histological analysis of selected cases, it limits the strength of the conclusions and the ability to generalize the outcomes. Further prospective studies with larger sample sizes and comparative controls are necessary to validate the clinical efficacy of the WHTPS technique.

### 2.4. Ethics Statement

This study was approved by the Institutional Review Board of Daegu Catholic University Medical Center (IRB No. 2025-04-011-001) and conducted in accordance with the ethical standards of the Declaration of Helsinki.

All patients signed a standardized written surgical consent form prior to treatment, which included explicit permission to collect tissue biopsies during surgery and to use anonymized clinical, radiographic, and histological data for academic research and publication. Core biopsies were collected using trephine burs from the planned implant sites before drilling and were integrated into the surgical workflow without additional clinical risk.

## 3. Case Presentation

### 3.1. Case 1

A 48-year-old male patient was referred from a private clinic for the reconstruction of severe vertical bone resorption in the right maxillary posterior region. The patient had no systemic medical conditions.

**Figure 1:**
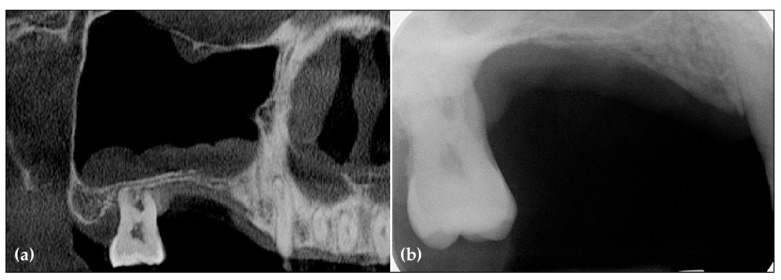
(**a**) The panoramic image and CBCT scans revealed severe vertical bone resorption along with a hopeless third molar. Additionally, a retention cyst was observed in the maxillary sinus; (**b**) The preoperative periapical radiograph revealed severe vertical ridge deficiency.

Preoperative periapical radiographs and CBCT scans revealed a hopeless second molar and significant horizontal and vertical bone deficiencies in the right posterior edentulous ridge. Additionally, a retention cyst was observed in the maxillary sinus (Figure 1).

**Figure 2:**
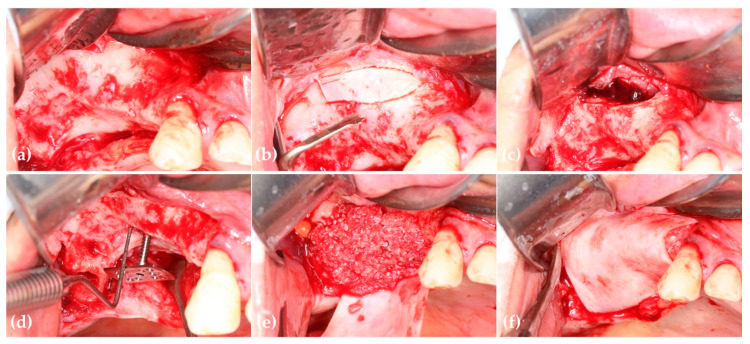
(**a**) Severe vertical deficiency in the right posterior maxilla; (**b**) The replaceable osteoinductive bony window (ROBW) was prepared using a thin-bladed saw tip connected to a piezoelectric device.; (**c**) Concentrated growth factor (CGF) membranes were placed beneath the elevated and perforated sinus membrane after removal of the retention cyst; (**d**) A rectangular wide-head tenting pole screw (WHTPS) was placed at the center of the defect to resist soft tissue contraction and support the graft; (**e**) Sticky autogenous tooth bone was grafted over the screw head to fill the defect. (**f**) A resorbable collagen barrier was placed over the graft to promote guided bone regeneration.

The surgical procedure was performed under local anesthesia following the intravenous administration of preoperative antibiotics (Flomoxef, Flumarin^®^, Ildong Pharm, Seoul, Republic of Korea). Venous blood was drawn from the patient’s forearm to produce autologous fibrin glue and concentrated growth factor (CGF) membranes, used to prepare sticky bone, as originally introduced by Sohn et al. [24]. After extracting the second molar, a crestal incision was made slightly shifted toward the palatal side. To ensure sufficient buccal flap elevation, a vertical incision was extended from the distal line angle of the canine to the oral mucosa, followed by a posterior incision at the end of the alveolar crest. A full-thickness buccal flap was elevated to expose the lateral wall of the maxillary sinus, and the palatal flap was also elevated to create space for grafting. All soft tissue from the bony defect was removed using a surgical curette. A piezoelectric bone surgery device (Surgybone; Silfradent srl, Sofia, Italy) with a saw tip was used to create a replaceable osteoinductive bony window (ROBW) on the lateral wall of the sinus. The anterior vertical osteotomy was placed 2 mm distal to the anterior wall of the maxillary sinus, and the inferior osteotomy 2 mm above the sinus floor. The vertical osteotomy was approximately 10 mm in height, with both osteotomies beveled toward the inner surface of the sinus wall. After carefully detaching the ROBW from the sinus mucosa, the sinus membrane was elevated using an elevator. A small incision was made in the membrane using a 15C blade, and a suction device was used to aspirate the cyst contents. Three CGF membranes were placed beneath the elevated membrane to promote healing of the perforated sinus mucosa. The ROBW was repositioned over the lateral bony window. A 1.2 mm pilot drill was used to prepare the insertion site for a rectangular WHTPS, which measured 14 mm in length, 6 mm in width, and 14 mm in height at the deepest area of the graft site. The screw was inserted at 50 rpm using a 0.048-inch hex driver connected to an implant handpiece. The head of the screw was positioned at the distal marginal ridge level of the canine. The extracted second molar was processed into osteoinductive autogenous tooth bone using the Vacuasonic machine (CosmoBioMedicare, Hanam-si, Gyeonggi-do, Republic of Korea). This tooth bone was mixed with autologous fibrin glue to form sticky bone and was grafted onto the alveolar ridge, covering the screw head. A 30 × 40 mm resorbable collagen membrane (Lysogide^®^, Oscotec Inc., Seongnam-si, Gyeonggi-do, Republic of Korea) was placed beneath the palatal flap to cover the grafted site. No membrane tacks or stabilization sutures were used. A periosteal releasing incision was performed on the buccal side, and the flap was sutured without tension. Due to the cohesive nature of the sticky bone and tension-free primary closure, the collagen membrane was stable without the need for additional fixation using screws or tacks (Figure 2).

**Figure 3:**
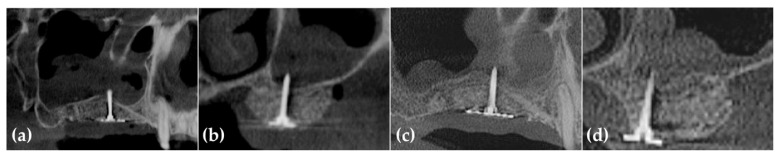
(**a**) Post-operative panoramic image of CBCT shows vertically grafted bone; (**b**) Cross-sectional images of CBCT reveal horizontally grafted bone; (**c**) Panoramic image of CBCT after 6 months of healing demonstrates that vertical alveolar ridge augmentation is distinctly observed; (**d**) The cross-sectional image at 6 months post-surgery demonstrates that horizontal ridge augmentation was remarkably well achieved.

**Figure 4:**
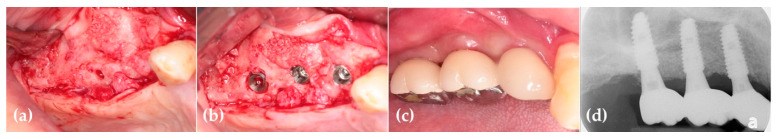
(**a**) After 6 months of healing, the WHTPS was removed, demonstrating excellent alveolar ridge regeneration and reconstruction. A bone biopsy was performed to assess tissue regeneration; (**b**) Three implants were placed; (**c**) Intraoral photographs taken 6 months after the final prosthesis placement showed stable soft tissue around the restoration (Restoration by Dr. Siwoo Lee); (**d**) Radiographic images taken 6 months after functional loading revealed stable crestal bone preservation above the implant platform. The letter a indicates the orientation mark of the radiograph.

Six months postoperatively, radiographs and CBCT showed successful vertical ridge augmentation. Upon re-entry, the buccal flap was reflected, and the WHTPS was removed, revealing satisfactory bone formation. To evaluate histologic outcomes, a bone biopsy was taken using a 2 mm inner diameter trephine bur beneath the screw head. Subsequently, three implants (Biotem Implant, Biotem Co., Hanam-si, Gyeonggi-do, Republic of Korea) measuring 10 mm in length and 4.5 mm in diameter were placed (Figure 3 and Figure 4).

**Figure 5:**
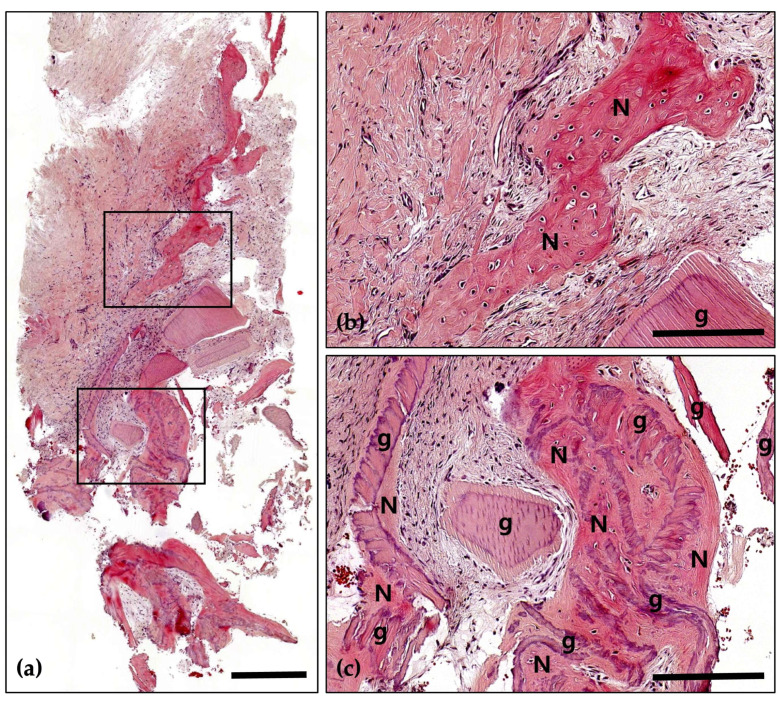
Hematoxylin and Eosin (H&E) staining; (**a**) Magnification 12.5×. The histological findings demonstrate successful graft integration, active osteogenesis, and a stable healing process. Notably, there are no signs of adverse inflammatory reactions or inflammation, indicating a favorable healing environment and effective bone regeneration (Scale bar: 500 μm). Black frames indicate the region of interest selected for higher magnification views in panels (**b**,**c**); (**b**) Magnification 100×—upper panel. Newly formed bone (N) is distinctly visible with well-defined osteocyte lacunae, signifying active bone formation and maturation. The residual graft material (g) is observed in close contact with the newly formed bone, indicating successful integration and demonstrating its osteoconductive properties (Scale bar: 100 μm); (**c**) Magnification 100×—lower panel. Newly formed bone (N) is prominently visible, characterized by well-defined osteocyte lacunae, which reflect active bone formation and maturation. The residual graft material (g) is closely associated with the newly formed bone, indicating successful integration and demonstrating its osteoconductive properties (Scale bar: 100 μm).

**Figure 6:**
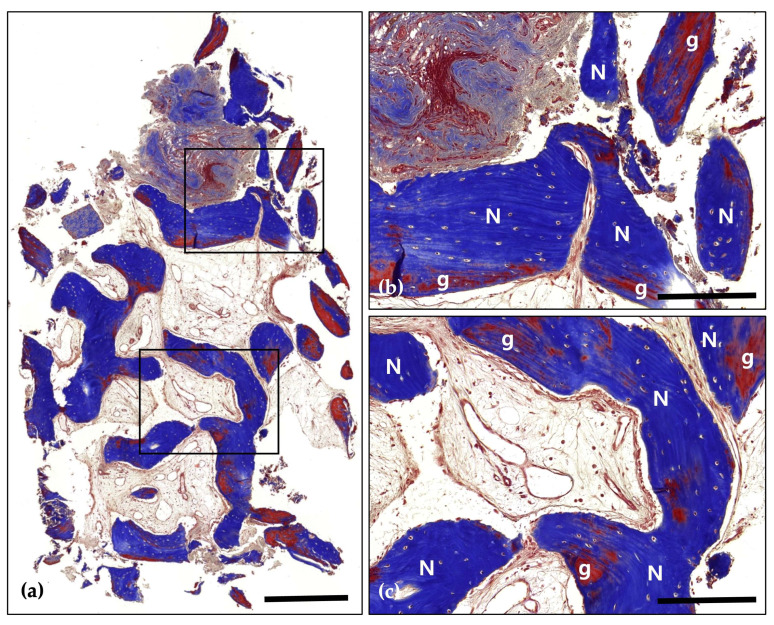
Masson’s Trichrome (MT) stain; (**a**) Magnification 12.5×. The distribution of new bone (blue-stained areas) around the bone grafts (red-stained areas) suggests effective graft incorporation and active bone regeneration (Scale bar: 500 μm). Black frames indicate the region of interest selected for higher magnification views in panels (**b**,**c**); (**b**) Magnification 100×—upper panel. Demonstrating direct contact between newly formed bone (N) and bone grafts (g). Evidence of active osteogenesis is seen, with mineralized bone (blue) forming around the graft materials (red). The surrounding area shows well-developed connective tissue with no signs of inflammation (Scale bar: 100 μm); (**c**) Magnification 100×—lower panel. Clear boundaries between the mineralized new bone (N) and the graft materials (g) are observed, indicating successful remodeling and integration of the graft within the host bone (Scale bar: 100 μm).

Following a 4-month healing period, the patient received the final prosthesis from their referring dentist. After 6 months of functional loading, intraoral photographs and radiographs demonstrated stable peri-implant soft tissue and well-maintained bone volume above the implant platform. Histological analysis revealed 21.2% new bone formation, with robust bone observed surrounding the tooth-derived graft material and no evidence of inflammation (Figure 5 and Figure 6).

### 3.2. Case 2

A 40-year-old male patient was referred from another hospital due to the need for severe bone defect reconstruction in the left maxillary posterior region. The patient had no significant medical history.

**Figure 7:**
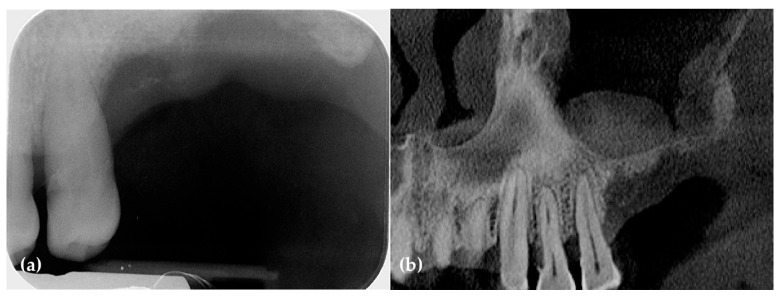
(**a**) Severe vertical bone defects are observed in the preoperative periapical radiograph; (**b**) The preoperative panoramic CBCT scan images also reveal significant vertical bone defects.

Preoperatively, periapical radiographs and CBCT panoramic images revealed a severe vertical bone defect and a mucosal retention cyst within the maxillary sinus (Figure 7).

Under local anesthesia, alveolar ridge reconstruction was performed following the same general protocol described in Case 1, including full-thickness flap elevation on both buccal and palatal sides and preparation of the WHTPS insertion site using a 1.2 mm drill at 50 rpm.

**Figure 8:**
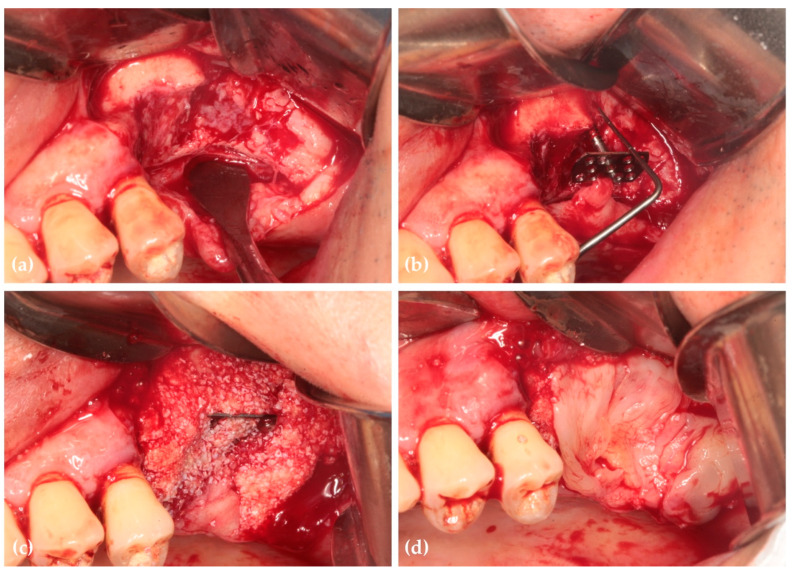
(**a**) After elevating the buccal flap, a severe vertical bone defect and poor bone quality were observed in the extraction socket; (**b**) To achieve initial stabilization of the WHTPS, it was placed into the buccal cortical bone with good bone quality. Notably, a 6 mm vertical defect was observed; (**c**) Sticky conditioned bone was grafted into the defect area; (**d**) After covering the bone graft material with a resorbable barrier membrane, four CGF membranes were placed over the barrier membrane to promote soft tissue healing. The soft tissue was then sutured without tension.

In this case, the bone quality at the extraction socket was notably poor due to incomplete healing. A rectangular WHTPS (12 mm length, 7 mm width, 10 mm thread height) was placed and adjusted to match the bone level of the adjacent second premolar, resulting in approximately 6 mm of vertical bone augmentation. Unlike Case 1, sticky bone was prepared using a 2:1 mixture of putty allograft (Accel, Ossgen Co., Daegu, Republic of Korea) and bovine bone (Bio-B™, Surgident Co., Daegu, Republic of Korea). The graft was applied in sufficient volume to fully cover the WHTPS, and a resorbable collagen membrane was placed over it without fixation. To further enhance soft tissue healing, four CGF membranes were applied over the grafted area. The flap was closed without tension using a periosteal releasing incision on the buccal side (Figure 8).

**Figure 9:**
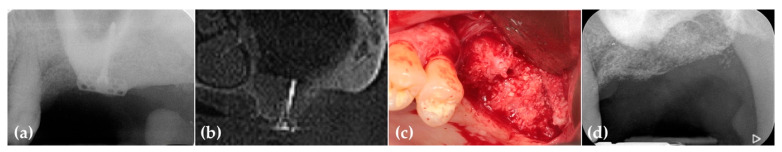
(**a**) Vertically grafted bone is observed around the WHTPS; (**b**) Cross-sectional CBCT image showing vertically grafted bone; (**c**) After 5 months of healing, the bone builder was removed, and remarkable three-dimensional bone regeneration was observed beneath the bone builder; (**d**) Periapical radiograph taken after the removal of the bone builder at 5 months shows sufficient vertical ridge augmentation. The triangle indicates the orientation marker of the periapical radiograph.

Five months later, the WHTPS was removed, revealing remarkable alveolar ridge reconstruction. Radiographic periapical images also confirmed sufficient 3-dimensional ridge augmentation. The patient returned to his dentist for implant placement (Figure 9).

**Figure 10:**
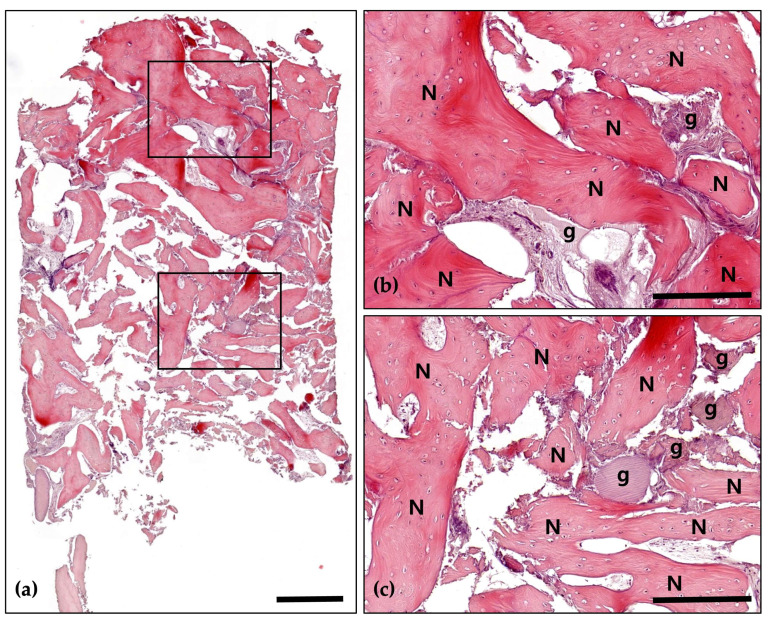
Hematoxylin and Eosin (H&E) staining; (**a**) Magnification 12.5×. A 29.6% rate of new bone formation was observed, with no signs of inflammation (Scale bar: 500 μm). Black frames indicate the region of interest selected for higher magnification views in panels (**b**,**c**); (**b**) Magnification 100×—upper panel. A significant amount of newly formed bone (N) is observed. Some newly formed bone is associated with a small amount of bone graft material (g), though the boundaries between them are indistinct under H&E staining. Active bone formation is prominent, with newly formed bone fragments visible. Sparse connective tissue is observed in the surrounding area (Scale bar: 100 μm); (**c**) Magnification 100×—lower panel. A considerable amount of newly formed bone (N) is observed in association with a small amount of allograft (g). While the new bone appears well-developed, the amount of connective tissue is insufficient compared to the newly formed bone. No signs of inflammation are present (Scale bar: 100 μm).

**Figure 11:**
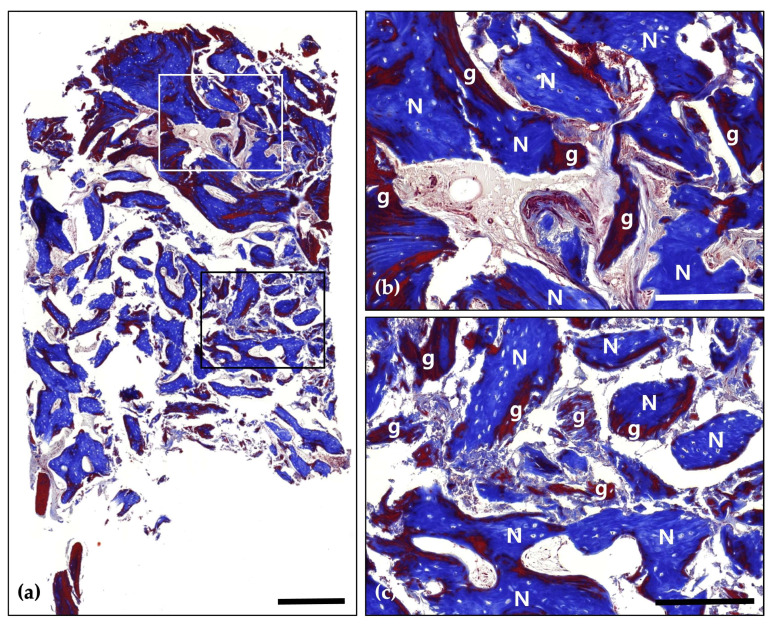
Masson’s Trichrome (MT) stain; (**a**) Magnification 12.5×. Active new bone formation (blue-stained) surrounds red-stained bone grafts, indicating robust bone regeneration (Scale bar: 500 μm). White, Black frames indicate the region of interest selected for higher magnification views in panels (**b**,**c**); (**b**) Magnification 100×—upper panel. Demonstrating direct contact between newly formed bone (N) and bone grafts (g). Evidence of active osteogenesis is seen, with mineralized bone (blue) forming around the graft materials (red). The amount of connective tissue is insufficient compared to the newly formed bone. No signs of inflammation are present (Scale bar: 100 μm); (**c**) Magnification 100×—lower panel. The lower panel shows clear integration between the graft material (g) and newly formed bone (N). Mineralized bone tissue (blue-stained) is observed connecting seamlessly with areas of the bone grafts (red-stained), highlighting effective graft incorporation and bone remodeling (Scale bar: 100 μm).

The histomorphometric analysis revealed vigorous new bone formation in the grafted area, with no signs of inflammatory reaction. Some of the newly formed bone was associated with residual graft materials, though the boundaries between the graft materials and the new bone appeared indistinct under H&E staining. This suggests successful integration of the graft material into the regenerating bone matrix. Newly formed bone accounted for 29.6% of the total area (Figure 10 and Figure 11).

### 3.3. Case 3

**Figure 12:**
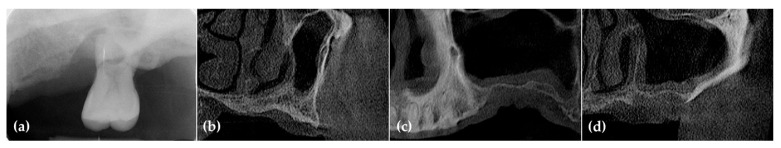
(**a**) A plain radiograph showed severe periodontal bone loss in the second molar; (**b**) The cross-sectional image of the preoperative CBCT scans revealed horizontal deficiency in the premolar area; (**c**,**d**) The panoramic image and cross-sectional CBCT scans demonstrate a severe three-dimensional bone defect.

A 48-year-old healthy male patient was referred from a private dental clinic for the management of significant vertical bone loss in the left maxillary posterior region. Preoperative periapical radiographs revealed a hopelessly compromised maxillary second molar with extensive periodontal destruction. The second molar was extracted and processed into autogenous tooth bone graft material using a Vacuasonic machine. Cross-sectional CBCT images showed a very narrow alveolar ridge in the premolar region, indicating substantial bone loss. Additionally, panoramic CBCT images demonstrated severe vertical resorption of the alveolar ridge in the posterior region, emphasizing the extent of the defect (Figure 12).

To address the severity of the bone deficiency, a combined surgical approach was planned. This included maxillary sinus augmentation using CGF blocks and simultaneous horizontal and vertical ridge augmentation. Sticky tooth bone graft material was utilized in combination with round and rectangular bone builders to maintain the structure of the graft and prevent its collapse due to the contraction of the soft tissue matrix.

The surgical procedure was performed under local anesthesia with antibiotic prophylaxis, as described in Case 1. Following full-thickness flap elevation and collecting venous blood for CGF and fibrin glue preparation, an ROBW was created using a piezoelectric device, and access to the sinus membrane was achieved similarly to Case 1.

**Figure 13:**
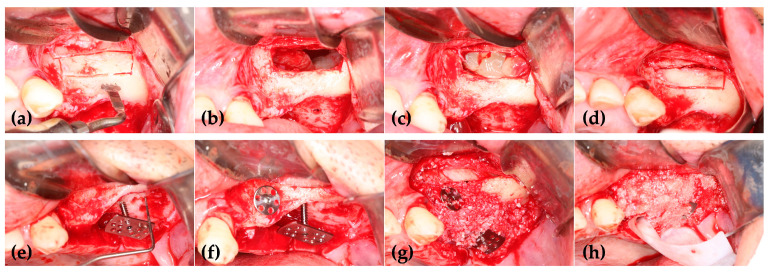
(**a**) Preparation of the ROBW; (**b**) Note the significant mucosal proliferation observed during the mucosal elevation process; (**c**) CGF blocks were placed under the perforated sinus mucosa to enhance bone regeneration and accelerate the healing of the perforated sinus mucosa; (**d**) Repositioning of the ROVW to maintain sinus integrity and accelerate bone regeneration in the sinus; (**e**) A rectangular WHTPS was placed at the center of the defect to facilitate new bone formation; (**f**) A round ROBW was placed on the buccal cortex to reconstruct horizontal deficiencies in the premolar region; (**g**) Sticky tooth bone was grafted to the defect site to promote bone regeneration and maintain the structure of the graft; (**h**) A collagen barrier was placed over the graft material to protect it and facilitate guided bone regeneration.

After cyst removal through a stab incision in the sinus membrane, the space was filled with four CGF blocks to promote healing. The ROBW was repositioned to act as a barrier. Vertical augmentation in the molar region was achieved using a rectangular WHTPS (6 mm × 10 mm), and a round WHTPS (6 mm diameter, 10 mm length) was placed in the premolar region for horizontal reconstruction. A sticky tooth bone graft was used to fill the defect, and in areas with insufficient volume, a mixture of putty allograft (Accel, Ossgen Co., Daegu, Republic of Korea) and porcine bone (BONE-XP, MedPark Co., Seoul, Republic of Korea) was used to form sticky conditioned bone. A collagen membrane was placed, and the flap was sutured without tension. Healing was uneventful before uncovering (Figure 13).

**Figure 14:**
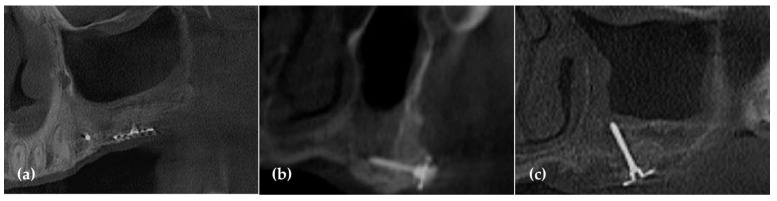
(**a**) The panoramic image of the CBCT after 5 months of healing shows successful vertical ridge augmentation and maxillary sinus augmentation; (**b**) Remarkable horizontal ridge augmentation is observed in the premolar region; (**c**) The cross-sectional CBCT image demonstrates both horizontal and vertical ridge augmentation.

**Figure 15:**
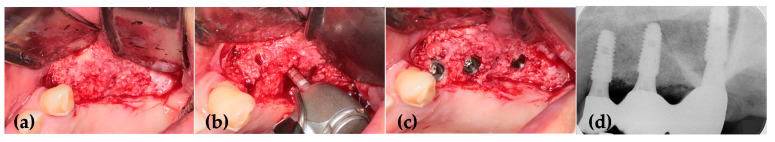
(**a**) After 5 months of healing, the WHTPSs were removed, revealing excellent bone regeneration; (**b**) A bone biopsy was performed to confirm the formation of newly generated bone; (**c**) Three implants were placed; (**d**) A radiograph taken 6 months after functional loading showed stable alveolar bone around the implants. (restoration; Dr Siwoo Lee).

After 5 months of healing, uncovering was performed, and CBCT scans demonstrated successful three-dimensional ridge augmentation around the rectangular WHTPS and horizontal reconstruction surrounding it. A bone biopsy using a 2 mm-wide trephine bur was performed to assess the degree of new bone formation in the reconstructed area (Figure 14 and Figure 15).

**Figure 16:**
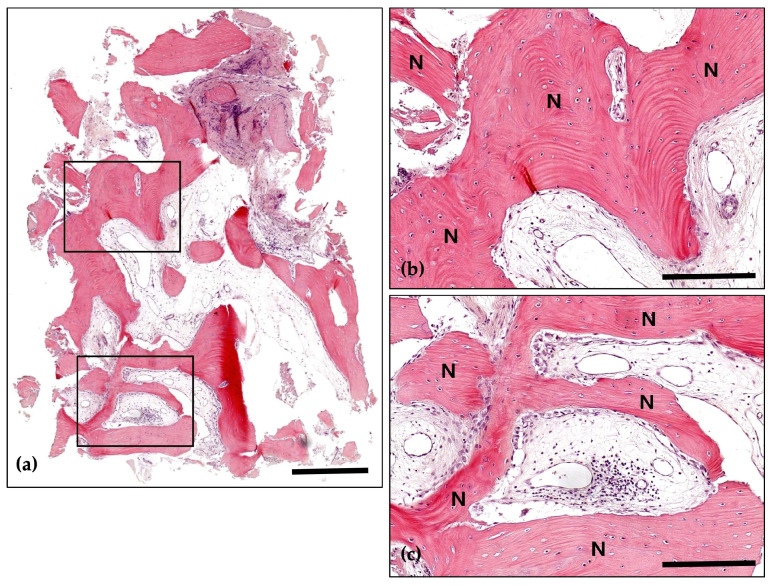
Hematoxylin and Eosin (H&E) staining; (**a**) magnification 12.5×. The histological section shows newly formed bone distributed throughout the defect site (Scale bar: 500 μm). Black frames indicate the region of interest selected for higher magnification views in panels (**b**,**c**); (**b**) Magnification 100×—upper panel. Newly formed bone (N) is evident with well-organized lamellar structures, indicating maturation of the bone matrix. Osteocyte lacunae are clearly visible within the bone, reflecting active bone remodeling and stability. The absence of inflammatory cells suggests a healthy healing process and successful regeneration (Scale bar: 100 μm); (**c**) Magnification 100×—lower panel. A similar pattern of newly formed bone (N) is observed, characterized by a dense bone matrix and clearly defined osteocyte lacunae. The seamless integration of the newly formed bone into the surrounding areas suggests effective and successful bone regeneration. The surrounding connective tissue is well-developed and rich in collagen fibers, and no signs of inflammation are observed (Scale bar: 100 μm).

**Figure 17:**
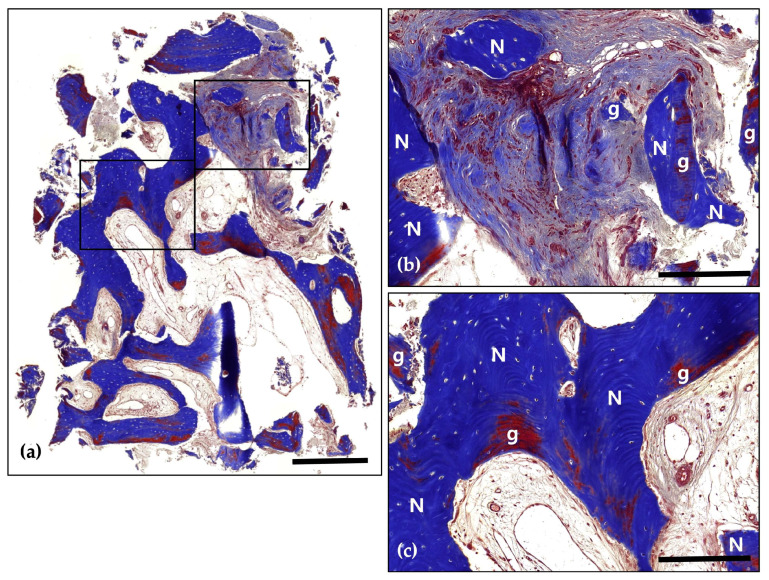
Masson’s Trichrome (MT) stain; (**a**) Magnification 12.5×. Active bone reformation is evident, with blue-stained newly formed bone surrounding red-stained osteoid tissue and graft material (Scale bar: 500 μm). Black frames indicate the region of interest selected for higher magnification views in panels (**b**,**c**); (**b**) Magnification 100×—upper panel. The histologic image demonstrates areas of active bone remodeling. Blue-stained bone (mineralized tissue) surrounds the red-stained graft, showing good integration (Scale bar: 100 μm); (**c**) Magnification 100×—lower panel. It highlights the close interaction between newly formed bone (N, blue-stained) and graft material (g, red-stained). Active remodeling is observed, with osteoid formation and evidence of osteoblast and osteoclast activity contributing to bone maturation (Scale bar: 100 μm).

Histomorphometric analysis revealed active new bone formation around the graft material, with no signs of inflammation observed. The graft material appears well-integrated with the surrounding bone, showing active new bone formation around its margins. The balance between mineralized bone and osteoid suggests a healthy bone regeneration process with no inflammatory response. The uniform distribution of new bone around the graft material highlights the success of the graft in promoting bone healing. The extent of new bone formation was measured at 32% (Figure 16 and Figure 17).

### 3.4. Case 4

A 47-year-old male patient was referred to our clinic from another dental practice solely for tooth extraction and subsequent alveolar ridge augmentation involving the left mandibular first and second premolars, as well as the second molar, which showed severe periodontal destruction. A comprehensive periodontal evaluation was performed before extraction at our institution, based on the classification criteria described in Section 2.1.2 of Section 2. The affected teeth demonstrated Grade II or higher mobility, deep periodontal pockets, and pronounced vertical bone loss on radiographic examination. Based on these findings, the teeth were diagnosed with a hopeless prognosis and were deemed irrational to treat with periodontal therapy alone. Tooth extraction was performed on the day of the initial visit. The extracted teeth were processed to prepare an autogenous tooth block bone and particulate autogenous tooth bone graft using the Vacuasonic machine one day before surgery.

**Figure 18:**
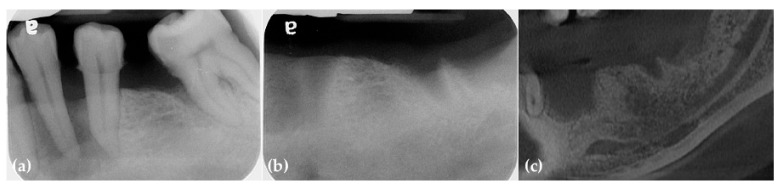
(**a**) Severe bone loss caused by advanced periodontal disease is observed around the first and second premolars; (**b**) The preoperative radiograph reveals severe vertical bone loss due to advanced periodontal disease following extraction; (**c**) The preoperative panoramic CBCT scan demonstrates significant vertical bone defects and extraction socket defects. The inverted letter a indicates the orientation mark of the radiograph.

After 6 weeks of soft tissue healing, the ridge augmentation procedure was performed under local anesthesia. Severe vertical bony defects were observed in the premolar region due to periodontitis on the preoperative radiograph. Preoperative CBCT images also revealed significant vertical bone loss along with alveolar bone defects in the extraction socket (Figure 18).

A crestal incision and a vertical release were performed, as described in Case 1. Full-thickness buccal and lingual flaps were elevated. The lingual flap was further dissected from the floor of the mouth using a periosteal elevator to release the mylohyoid muscle attachment. The flap and periosteum were carefully separated in both lingual and crestal directions to ensure adequate mobilization and tension-free closure. The lingual flap was then retracted and stabilized to the contralateral side using a 4-0 nylon suture to maintain surgical visibility. After complete removal of granulation tissue from the premolar extraction socket, a severe vertical ridge defect was noted. An osteotomy was created using a 1.2 mm pilot drill, and a 6 mm diameter, 12 mm length round WHTPS was placed at 50 rpm. The head height was adjusted to match the level of the lingual crest, thereby supporting the graft against soft tissue contraction. To augment the horizontal defect in the posterior region, a tooth block was fixed to the buccal cortical bone using a mini-screw. Additionally, sticky particulate tooth bone was grafted into the vertical defect. Instead of a collagen membrane, three CGF membranes were layered over the graft to support wound healing.

**Figure 19:**
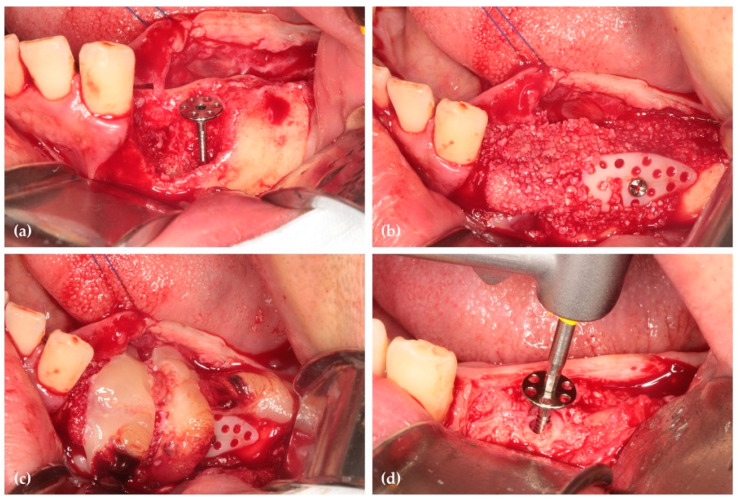
(**a**) Note the severe vertical defect in the extraction site after elevating the anterior flap. A 6 mm diameter round WHTPS was placed at the bony defect site to prevent the collapse of the bone graft material from the compression of the soft tissue matrix during the healing period; (**b**) For horizontal ridge augmentation of the narrow alveolar ridge in the mandibular posterior region, a tooth block bone graft was placed and stabilized using a mini-screw. In the maxillary posterior defect area, sticky tooth bone was grafted, extending beyond the cover screw of the WHTPS. (**c**) Instead of using a resorbable collagen membrane, three layers of CGF membranes were placed over the bone graft material to promote soft tissue healing. A tension-free closure was then achieved using a releasing incision and suturing the soft tissue without tension; (**d**) Remarkable three-dimensional ridge augmentation was observed after 5 months of healing.

**Figure 20:**
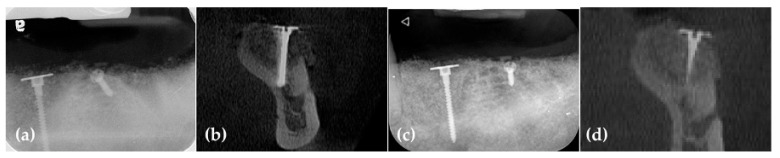
(**a**) A postoperative radiograph shows the grafted bone material placed over the WHTPS; (**b**) A cross-sectional CBCT image taken immediately after surgery reveals the grafted bone material surrounding the bone builder; (**c**) At 5 months postoperative, a radiograph shows that the grafted bone material remains well-maintained without collapse, extending up to the cover screw of the WHTPS; (**d**) Cross-sectional CBCT images at 5 months of healing demonstrate that the grafted bone material surrounding the bone builder remains intact. The inverted letter a and the triangle indicate orientation marks of the radiograph.

After five months of healing, the WHTPS and mini-screw were removed, revealing excellent outcomes in both vertical and horizontal ridge augmentation. A bone biopsy was performed to evaluate bone regeneration histomorphometrically. Following the soft tissue closure, the patient was referred back to their dentist for implant placement (Figure 19 and Figure 20).

**Figure 21:**
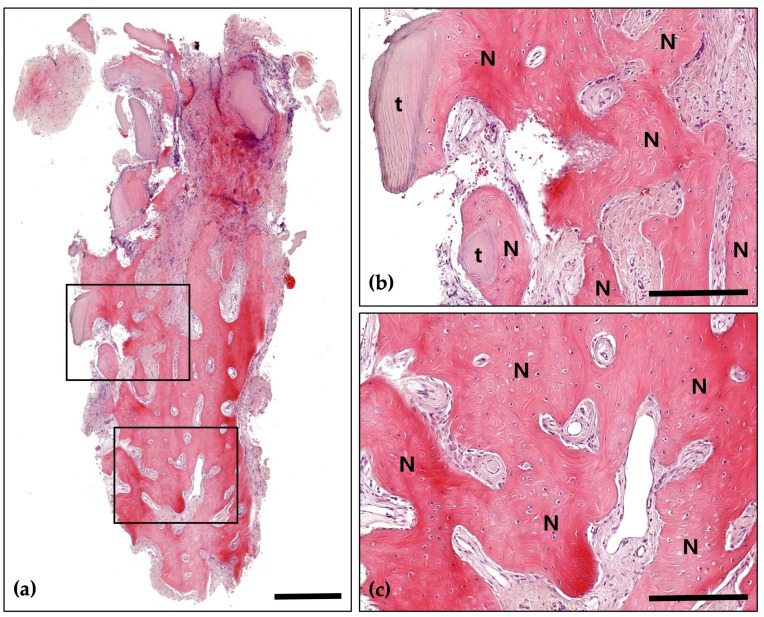
Hematoxylin and Eosin staining; (**a**) Magnification 12.5×. Histologic findings reveal active new bone formation surrounding tooth bone grafts. No inflammation is observed (Scale bar: 500 μm). Black frames indicate the region of interest selected for higher magnification views in panels (**b**,**c**); (**b**) Magnification 100×—upper panel. Straightforward integration of residual tooth graft material (t) with newly formed bone (N). The graft material is surrounded by mineralized new bone, indicating active bone regeneration and successful graft incorporation. The newly formed bone demonstrates visible osteocytes embedded within the bone matrix, indicating healthy bone maturation and remodeling. On the surface of the new bone, osteoblasts are prominently observed, reflecting active bone formation and deposition. Importantly, no signs of inflammation are present, suggesting a stable and favorable healing environment with no immune or inflammatory response interfering with the regenerative process. This indicates successful integration and regeneration of the graft material into functional bone tissue (Scale bar: 100 μm); (**c**) Magnification 100×—lower panel. The newly formed bone (N) demonstrates distinct osteocytes within the bone matrix, indicating proper maturation and integration of the bone. On the surface of the new bone, osteoblasts are visible, suggesting ongoing bone formation and remodeling. Notably, no graft material is observed in these areas, which indicates either complete resorption or integration of the graft material into the regenerated bone. Additionally, the absence of inflammatory signs confirms a stable healing process, free from adverse immune reactions. This reflects a successful regenerative outcome with functional and healthy bone tissue (Scale bar: 100 μm).

**Figure 22:**
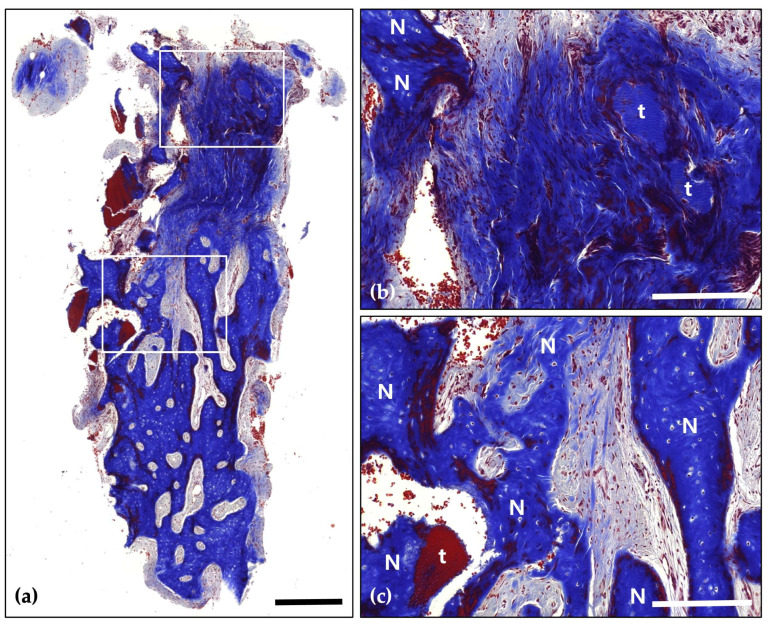
Masson’s Trichrome (MT) stain; (**a**) Magnification 12.5×. The histological section shows mineralized new bone (blue), residual tooth bone graft material (red), and surrounding connective tissue. Numerous collagen fiber bundles are present, with graft material visible among them. A small amount of newly formed bone is observed surrounding the graft material (Scale bar: 500 μm). White frames indicate the region of interest selected for higher magnification views in panels (**b**,**c**); (**b**) Magnification 100×—upper panel. The upper panel highlights the interaction between residual tooth graft material (t) and newly formed bone (N). Evidence of bone deposition around the graft material demonstrates successful incorporation and bone regeneration (Scale bar: 100 μm); (**c**) Magnification 100×—lower panel. The lower panel reveals active new bone formation (N) within abundant connective tissue. Even with minimal tooth graft material (t), prominent new bone formation is observed. Newly formed bone contains distinct osteocytes, and osteoblasts are clearly visible on its surface, indicating active regeneration (Scale bar: 100 μm).

Histological evaluation revealed active new bone regeneration, with newly formed bone bridging the gaps around the tooth bone graft material. The highlighted regions demonstrated significant bone remodeling and integration between the newly formed bone and graft particles. The newly formed bone exhibited a trabecular structure, indicative of ongoing osteogenesis and successful integration into the surrounding tissue. Remarkably, despite the short healing period of only 5 months, 35.9% new bone regeneration was observed, independent of whether a collagen membrane was used (Figure 21 and Figure 22).

### 3.5. Case 5

A 58-year-old male patient with complete tooth loss and moderate to severe alveolar bone resorption throughout the mandible presented to our clinic seeking implant placement and alveolar bone reconstruction. The patient had a medical history of hypertension, cerebral infarction, hyperlipidemia, and liver disease and was under the care of the departments of neurosurgery and gastroenterology at Daegu Catholic University Medical Center. He was also taking an antiplatelet agent.

**Figure 23:**
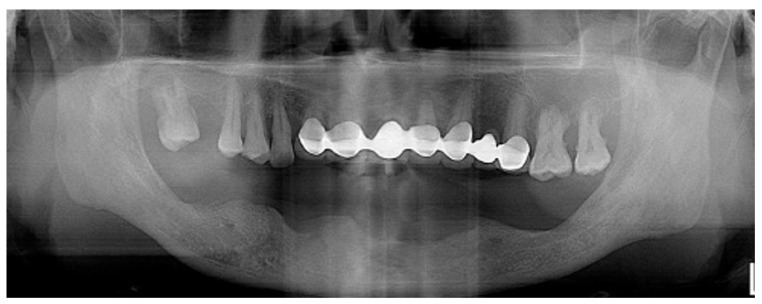
The preoperative panoramic radiograph reveals generalized moderate to severe alveolar bone resorption in the mandible, with particularly pronounced bone defects observed in the left posterior region.

Since the patient was completely edentulous in the posterior mandible, a periodontal evaluation could not be performed. Periodontal parameters such as probing depth, tooth mobility, and clinical attachment level require the presence of teeth and were therefore not applicable. However, a preoperative panoramic radiograph revealed generalized moderate to severe alveolar bone resorption, with pronounced vertical bone defects in the posterior edentulous ridge areas on both sides due to advanced bone loss (Figure 23). Based on these radiographic findings, vertical ridge augmentation using a WHTPS was planned to reconstruct the deficient ridge in preparation for implant placement.

The surgical procedure was performed under local anesthesia following the intravenous administration of preoperative antibiotics, as described in Case 1. Autologous fibrin glue and CGF membranes were prepared from venous blood, as in Case 1, and used to create sticky bone.

**Figure 24:**
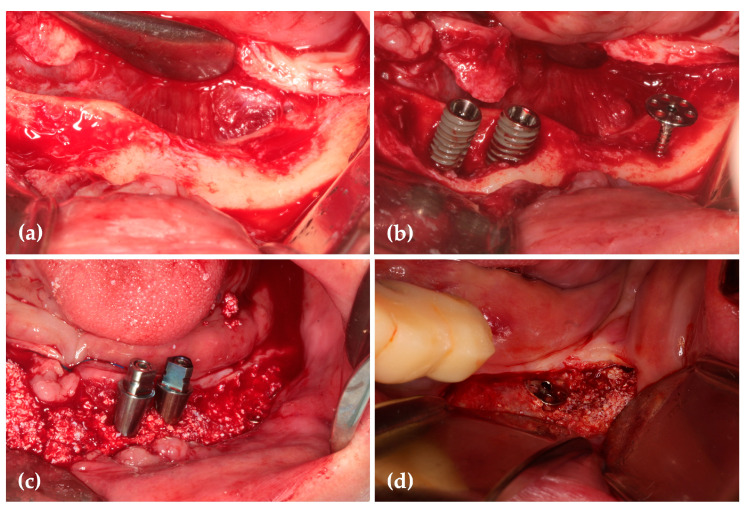
(**a**) After elevating the anterior flap, a severe vertical alveolar bone defect was observed in the left posterior region; (**b**) Dental implants were placed in the #33 and #34 regions, and a 6-mm diameter round WHTPS was installed at the site of the severe vertical alveolar bone defect posterior to these implants to prevent the collapse of the bone graft material caused by soft tissue pressure during the healing process; (**c**) A sticky bone graft material composed of a mixture of autogenous bone, allograft, and xenograft was placed around the implant sites and the WHTPS, extending beyond the head of the WHTPS; (**d**) A resorbable membrane was placed for GBR. Due to the sticky consistency of the graft, no additional fixation was necessary. CGF membranes were added to promote soft tissue healing.

A crestal incision was made with a 15C blade, extending forward to the mandible’s both retromolar pads. Vertical releasing incisions were added on both sides to allow for full-thickness elevation of the buccal flap, followed by elevation and release of the lingual flap through careful dissection of the periosteum and superficial mylohyoid fibers, as performed in Case 1. Five implants (Biotem Implant, Biotem Co., Hanam-si, Gyeonggi-do, Republic of Korea) were placed in the right posterior and bilateral canine areas. In the left posterior mandible, approximately 6 mm of vertical bone augmentation was needed due to a severe alveolar ridge defect. Autogenous bone was harvested from the buccal side of the right posterior mandible using an auto chip maker (ACM) drill (Osstem Implant Co., Seoul, Republic of Korea). The harvested bone was mixed with a putty-type allograft (Accel, Ossgen Co., Daegu, Republic of Korea), a bovine-derived xenograft (InduCera, Oscotec Inc., Seongnam-si, Gyeonggi-do, Republic of Korea), and autologous fibrin glue to form a cohesive, sticky graft. The composite graft material was applied around the WHTPS, which was placed at the defect site. A 30 × 40 mm resorbable collagen membrane was positioned over the graft, and CGF membranes were layered on top to further support soft tissue healing. Due to the cohesive nature of the sticky graft, no additional fixation, such as membrane tacks or stabilization sutures, was required. A periosteal releasing incision was made to allow for tension-free closure of the flap, which was then securely sutured (Figure 24).

**Figure 25:**
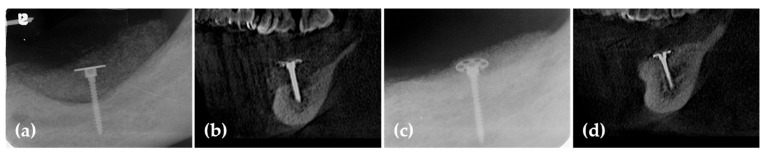
(**a**) A postoperative radiograph shows the grafted bone material placed over the WHTPS; (**b**) A cross-sectional CBCT image taken immediately after surgery reveals the grafted bone material surrounding the bone builder; (**c**) At 18 months postoperative, a radiograph shows that the grafted bone material remains well-maintained without collapse, extending up to the cover screw of the WHTPS; (**d**) Cross-sectional CBCT images at 18 months of healing demonstrate that the grafted bone material surrounding the bone builder remains intact. The inverted letter a indicates orientation mark of the radiograph.

Eighteen months after surgery, radiographic and CBCT scans showed successful three-dimensional ridge augmentation around the WHTPS (Figure 25). The buccal flap was elevated, and the WHTPS was removed, revealing satisfactory bone reconstruction. To assess the histological features of the newly formed bone beneath the wide head of the tenting pole screw, a bone biopsy was obtained using a trephine bur with a 3mm inner diameter.

**Figure 26:**
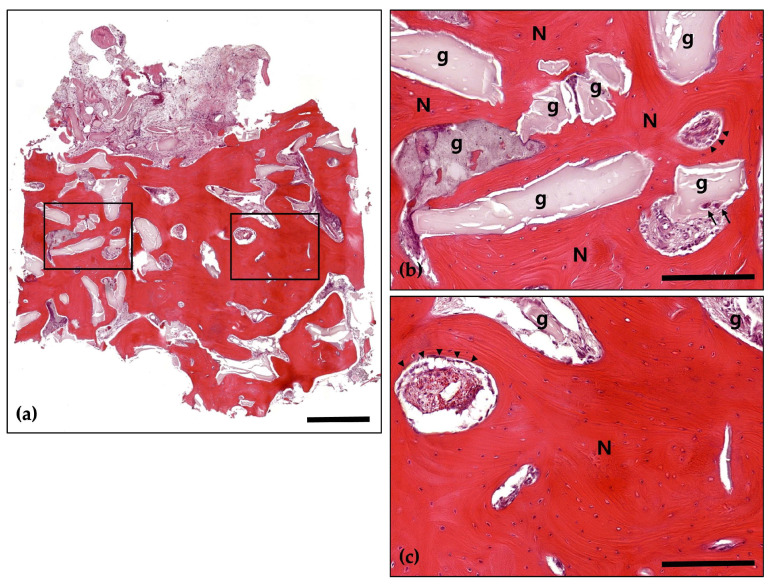
Hematoxylin and Eosin staining; (**a**) Magnification 12.5×. A 57.5% rate of new bone formation was observed, with no signs of inflammation. Overall, a well-developed bone structure is observed. A large amount of connective tissue and numerous graft materials are observed in the upper part, and new bone formation in this area is minimal. In the lower part of the well-developed mature bone, a bone marrow structure containing fat cells is observed (Scale bar: 500 μm). Black frames indicate the region of interest selected for higher magnification views in panels (**b**,**c**); (**b**) Magnification 100×—upper panel. Numerous graft materials (g) are observed in conjunction with well-developed bone (N). In some cases, graft material absorption by osteoclasts (arrows) is observed, and in some cases, new bone formation by osteoblasts (arrowheads) is also observed. In mature bone, numerous osteocytes are evident (Scale bar: 100 μm); (**c**) Magnification 100×—lower panel. A well-developed, mature bone (N) structure is observed. The amount of connective tissue is minimal, but in some cases, new bone formation by osteoblasts (arrowheads) is observed (Scale bar: 100 μm).

**Figure 27:**
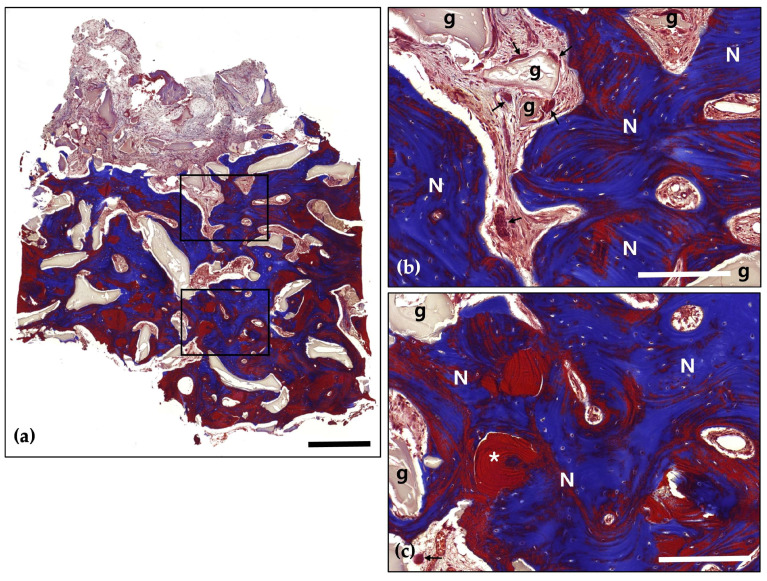
Masson’s Trichrome (MT) stain; (**a**) Magnification 12.5×. The structure of well-developed bone is observed. As a result of the MT stain, new bone (blue) and mature bone or lamellar bone (red) are observed separately (Scale bar: 500 μm). Black frames indicate the region of interest selected for higher magnification views in panels (**b**,**c**); (**b**) Magnification 100×—upper panel. The absorption of graft materials is observed between well-developed bones (N). Many osteoclasts (arrows) are observed in the connective tissue around the graft material (g). Most of the osteoclasts are located at the border with the graft material, showing active absorption of the graft material. Osteocytes are distinct in mature bone (Scale bar: 100 μm); (**c**) Magnification 100×—lower panel. Lamellar bone (asterisk) is distinct in the structure of well-developed bone (N). The bone matrix of mature bone contains distinct osteocytes, and osteoblasts are in small proportions. The absorption of graft materials (g) by osteoclasts (arrows) is observed in the connective tissue between the bones (Scale bar: 100 μm).

Histomorphometric analysis demonstrated partial presence of bone marrow structures containing adipocytes beneath the well-developed mature bone. Numerous osteoclasts were observed resorbing the graft material within the connective tissue between areas of mature bone and around the graft particles. In certain regions, active new bone formation by osteoblasts was evident, and osteocytes were identified within the mature bone. Under H&E staining, the interface between the graft material and the newly formed bone appeared indistinct, indicating successful integration of the graft material into the regenerating bone matrix. Newly formed bone occupied 57.5% of the total area, with no evidence of inflammatory response (Figure 26 and Figure 27).

In all five cases, radiographic, clinical, and histological assessments consistently confirmed that new bone formation extended vertically up to the level of the screw head (Table 1).

Overall, histomorphometric analysis across all five cases revealed consistent new bone formation, ranging from 21.2% to 57.5%. The newly formed bone was consistently observed in close contact with residual graft particles, and no inflammatory cell infiltration was noted in any of the specimens. These findings suggest that the graft materials used in conjunction with the wide-head tenting pole screw exhibit effective osteogenesis and favorable biocompatibility.

## 4. Discussion

Tooth loss continues to be a prevalent condition, often resulting in compromised mastication, changes in facial esthetics, and diminished quality of life [1]. The etiology includes traumatic avulsion, root fractures, dental caries, and advanced periodontal disease. Following tooth loss, the alveolar bone undergoes both horizontal and vertical resorption, most notably within the first 3–6 months, with continued atrophy reported even beyond three years [25].

Implant-supported removable or fixed prostheses restore masticatory function and aesthetics lost due to tooth loss, showing reliable success rates. Today, this treatment method is widely accepted as the standard of care [3].

However, severely resorbed alveolar bone defects in both the vertical and horizontal dimensions make it challenging to place implants in the desired position. This can lead to an unfavorable crown-to-implant ratio or improper masticatory force direction, negatively affecting the long-term prognosis of the implant. Additionally, it may pose a risk of damage to the surrounding anatomical structures [26].

Therefore, alveolar ridge augmentation is essential for the accurate placement of implants in severely resorbed alveolar bone. Various treatment modalities have been developed and are currently utilized to achieve this goal [27].

Implants placed in vertically augmented ridges generally show favorable outcomes, with reported success rates consistently exceeding 92–95% across different augmentation techniques, in line with systematic reviews and clinical studies that also noted relatively low rates of biological and technical complications [17,28,29,30].

Autogenous block bone grafting, which involves harvesting autogenous bone from donor sites such as the iliac crest or mandibular ramus and transplanting it to severely resorbed alveolar regions, is widely recognized as the gold standard. This approach is favored due to the osteoconductive, osteoinductive, and osteogenic properties of autogenous bone, along with its capacity for space maintenance [31,32].

However, autogenous block bone grafting requires bone harvesting from the donor site, which limits the available graft volume. It is also associated with prolonged surgical time, high costs, postoperative discomfort, and complications at both the donor and recipient sites. Specifically, this procedure is often associated with wound dehiscence, sensory disturbances, and infection at both donor and recipient sites, which can significantly affect patient morbidity and clinical predictability. Furthermore, the resorption rate remains unpredictable, posing a significant limitation to its clinical application [11,31,32,33,34,35,36].

To overcome the limitations of autogenous block bone grafting, alternative techniques such as distraction osteogenesis and sandwich osteotomy have been developed and are currently in use [37,38].

Distraction osteogenesis is a technique that involves a gradual expansion of a segmented bone using a distractor while facilitating bone grafting. Since the lingual or palatal soft tissues remain untouched, this method enables simultaneous augmentation of both hard and soft tissues while preserving an intact blood supply. Additionally, it eliminates the need for harvesting from other donor sites, providing a significant advantage [39]. However, its primary limitation is that it only supports vertical bone augmentation, necessitating additional bone grafting procedures when horizontal augmentation is required. Furthermore, the control over the distraction direction is limited, which may result in undesired bone elongation in unintended directions [40]. In addition to these limitations, distraction osteogenesis has been associated with complications such as fracture of the transport segment, soft tissue dehiscence, and infection, particularly in cases with inadequate soft tissue compliance or poor patient hygiene [14,41,42].

A modified form of distraction osteogenesis, the sandwich osteotomy, was developed for the anterior mandible. This technique combines horizontal alveolar osteotomy with pedicled bone grafting, offering the advantage of minimal resorption at the graft site. However, similar to distraction osteogenesis, it is limited in addressing horizontal deficiencies and often necessitates additional grafting procedures [43]. Sandwich osteotomy has also been associated with complications, including fracture of the transport segment and soft tissue dehiscence at the graft site, particularly in cases with inadequate stabilization or excessive movement during healing [16,17,44]. In contrast, autogenous split block bone techniques have shown predictable and stable three-dimensional vertical augmentation in the posterior maxilla, with high long-term success rates [45].

The aforementioned bone grafting techniques are further limited by their high surgical complexity, which contributes to significant variations in outcomes depending on the operator’s experience.

GBR was developed to overcome this surgical complexity. This technique involves placing a bone graft in the defect area and covering it with a membrane to prevent the infiltration of epithelial and gingival connective tissues while promoting bone formation within the membrane-covered space [46].

However, in guided bone regeneration, using resorbable membranes presents a challenge due to their insufficient mechanical strength. When soft tissue contracts, it becomes challenging to maintain space solely with the underlying bone graft material, which potentially may lead to graft displacement. To overcome this limitation, non-resorbable membranes or titanium mesh with greater mechanical strength can be utilized. While these materials are effective in maintaining space, they also increase the risk of membrane exposure, which increases the likelihood of contamination at the graft site. Furthermore, to prevent membrane displacement, bone tacks are often required, adding complexity to the procedure [47,48,49]. Clinically, GBR procedures have been associated with membrane exposure, graft contamination, and wound dehiscence, which can compromise regenerative results and require additional interventions [6,46,47,48].

The tent-pole technique was developed to overcome the limitations of guided bone regeneration. The tent-pole technique is a modified approach based on the principles of guided bone regeneration. This method uses mini-screws to elevate the periosteum and soft tissue like a tent, providing structural support to the membrane against compressive forces from soft tissue contraction while maintaining the stability of the grafted site [50].

The tent-pole screw technique presents a less invasive and technically more straightforward approach compared to other bone grafting methods, with reduced graft resorption. Additionally, it removes the necessity for autogenous bone harvesting and shows a lower complication rate, even when using a non-resorbable membrane [51].

However, the tent-pole screw technique has certain limitations. Since implants cannot be placed at the same time, it may lead to a prolonged edentulous period and an increased number of surgical procedures. Additionally, the support provided by a single screw is limited, requiring multiple screws for extensive bone defects [52].

The tent-pole implant technique, which utilizes implants as structural supports, was first introduced by Marx et al. in 2002 [53]. This approach involves an extraoral surgical method to augment a severely resorbed, fully edentulous mandible by placing implants in the anterior mandible and using them as a tent-pole. The advantage of this technique is that it uses implants to create a tent-pole effect, which provides stable support for the overlying soft tissue and enhances the stability of the surrounding bone graft material. As a result, it increases vertical alveolar bone height and width while reducing the risk of pathological mandibular fractures. Additionally, unlike the tent-pole screw technique, this method allows for simultaneous implant placement, thereby shortening the time needed for prosthetic rehabilitation and reducing the number of surgical procedures. However, this procedure must be conducted under general anesthesia, which can cause a physiological burden on the patient. Additionally, it is not suitable for partially edentulous patients or those with moderate or less severe alveolar bone resorption. In contrast, vertical ridge augmentation using tent-pole implants and abutments under local anesthesia has been reported as a predictable technique in partially edentulous patients [54].

However, in cases where vertical ridge augmentation is required but implants cannot be used as tent-pole supports, mini-screws are typically employed to maintain space. While effective, techniques using mini-screws have inherent limitations, as previously discussed. To overcome these drawbacks—particularly the need for multiple screws in large alveolar bone defects caused by severe resorption—a wide-head tenting pole screw was developed. This design provides enhanced support for the periosteum and surrounding soft tissue matrix, enabling reliable space maintenance and improving surgical outcomes, especially in pontic areas with severe vertical deficiencies between implants [21].

The wide-head tent-pole screw is designed with a broad, flat head to support stable expansion of the overlying soft tissue matrix. Micro-perforations on the head allow blood infiltration, which may facilitate early vascularization and bone regeneration.

Compared to conventional GBR, the WHTPS technique provided more predictable space maintenance without requiring fixation devices such as tacks or titanium meshes. While resorbable membranes in GBR are prone to collapse due to their limited mechanical strength, non-resorbable options, such as titanium meshes, although more stable, carry increased risks of exposure and infection [5,6,47,48]. The WHTPS design effectively resists soft tissue pressure, simplifying the procedure and eliminating the need for additional fixation [21,51]. Furthermore, recent clinical reports have provided comparative insights into other vertical ridge augmentation techniques. The use of titanium mesh has been associated with high rates of soft tissue dehiscence and graft site complications, particularly when applied in wide edentulous spans or under functional loading conditions [55]. GBR using pins improves membrane stability but frequently requires a second-stage surgery for pin removal and may lead to soft tissue irritation or inflammation around fixation points [56]. The dome technique offers reliable space maintenance using titanium caps; however, it may be limited by anatomical constraints and soft tissue tension [57]. In comparison, the WHTPS provides a simpler and less invasive alternative with reduced risk of mesh exposure and fewer surgical steps, while maintaining sufficient space and graft stability.

Autogenous block bone grafting is widely regarded as the gold standard for vertical ridge augmentation due to its osteogenic capacity and ability to maintain space. However, its application is limited by the need for donor site surgery, extended operative time, and the risk of unpredictable graft resorption. In our study, up to 57.5% vertical bone formation was achieved without the need for autogenous bone harvesting, thereby reducing surgical morbidity while maintaining graft stability and histologic integrity [31,32,33].

Osteogenesis-based techniques, such as distraction osteogenesis and sandwich osteotomy, also demonstrate favorable outcomes in vertical bone augmentation; however, they require advanced surgical skills and are associated with more extended healing periods and complications, including fracture, soft tissue dehiscence, and vector control limitations [14,16,17]. In contrast, the WHTPS technique offers a technically simpler, single-stage approach with fewer complications and consistent histological results across all cases [21,54].

In the present study, histomorphometric analysis revealed new bone formation ranging from 21.2% to 57.5%. These values align with previous literature, which reported mean new bone percentages of 32.98% ± 8.27% using allogeneic grafts and up to 43.5% ± 11.5% with autogenous bone combined with xenografts in vertical ridge augmentation procedures [58,59]. This comparison highlights the clinical relevance of the WHTPS technique in achieving effective osteogenesis.

In addition to these comparative advantages, the broader head of the WHTPS further enhances spatial stability in extensive vertical defects and distributes mechanical stress more evenly, minimizing the risk of graft displacement or collapse.

Building on these biomechanical and procedural benefits, the WHTPS technique demonstrated excellent clinical practicality in the present cases. The use of a single WHTPS in combination with sticky bone simplified the surgical procedure compared to conventional GBR techniques, reducing overall operative time. The approach was also cost-effective, as it eliminated the need for additional fixation devices or custom-made meshes. Notably, all five cases exhibited uneventful healing without complications such as wound dehiscence, graft exposure, or infection. Furthermore, no secondary surgical interventions were required, and satisfactory bone regeneration was achieved in all cases.

Among the five cases, follow-up data after prosthesis delivery were available up to 6 months for Case 1, during which no complications were observed, and peri-implant tissues remained stable.

These findings collectively demonstrate that the WHTPS technique reduces surgical time, improves cost-effectiveness, avoids the need for second interventions, and minimizes healing complications compared with conventional vertical ridge augmentation procedures.

### Limitations

Despite the favorable clinical and histological outcomes, this study has several inherent limitations. Long-term follow-up beyond one year was not available for any of the patients, and only short-term post-prosthetic data were recorded. Moreover, the study design, being a small retrospective case series without a comparator group, restricts the ability to draw definitive conclusions regarding the efficacy and safety of the WHTPS technique. Additionally, patient-reported outcome measures (PROMs) were not systematically collected, which limits the ability to assess patient-centered aspects, such as pain, satisfaction, and quality of life. Therefore, the present results should be interpreted as preliminary, and further prospective controlled trials with larger cohorts, long-term observation, and validated PROMs are required to validate and generalize these findings.

## 5. Conclusions

Vertical bone regeneration was consistently observed across all cases, as evidenced by clinical, radiographic, and histological assessments. The use of a single wide-head tenting pole screw appeared effective in maintaining space under soft tissue pressure, with new bone formation extending to the level of the screw head. However, as this was a retrospective case series with a small sample size and no control group, the findings should be interpreted as preliminary. Well-designed randomized controlled trials are necessary to further validate the efficacy and predictability of this technique.

## Figures and Tables

**Table 1:** Summary of vertical ridge augmentation outcomes using WHTPSs in five clinical cases.

Case No.	Location	Augmentation Height (mm)	Bone Formation (%)	Healing Duration Before Biopsy (Month)	Type of Graft Used
1	Maxilla Right	10 mm	21.2%	6	Autogenous tooth bone
2	Maxilla Left	6 mm	29.6%	5	Allograft + Xenograft
3	Maxilla Left	10 mm	32.0%	5	Allograft + Xenograft
4	Mandible Left	9 mm	35.9%	5	Autogenous tooth bone
5	Mandible Right	8 mm	57.5%	18	Autograft + Allograft + Xenograft

## Data Availability

The data presented in this study are available on request from the corresponding author.
